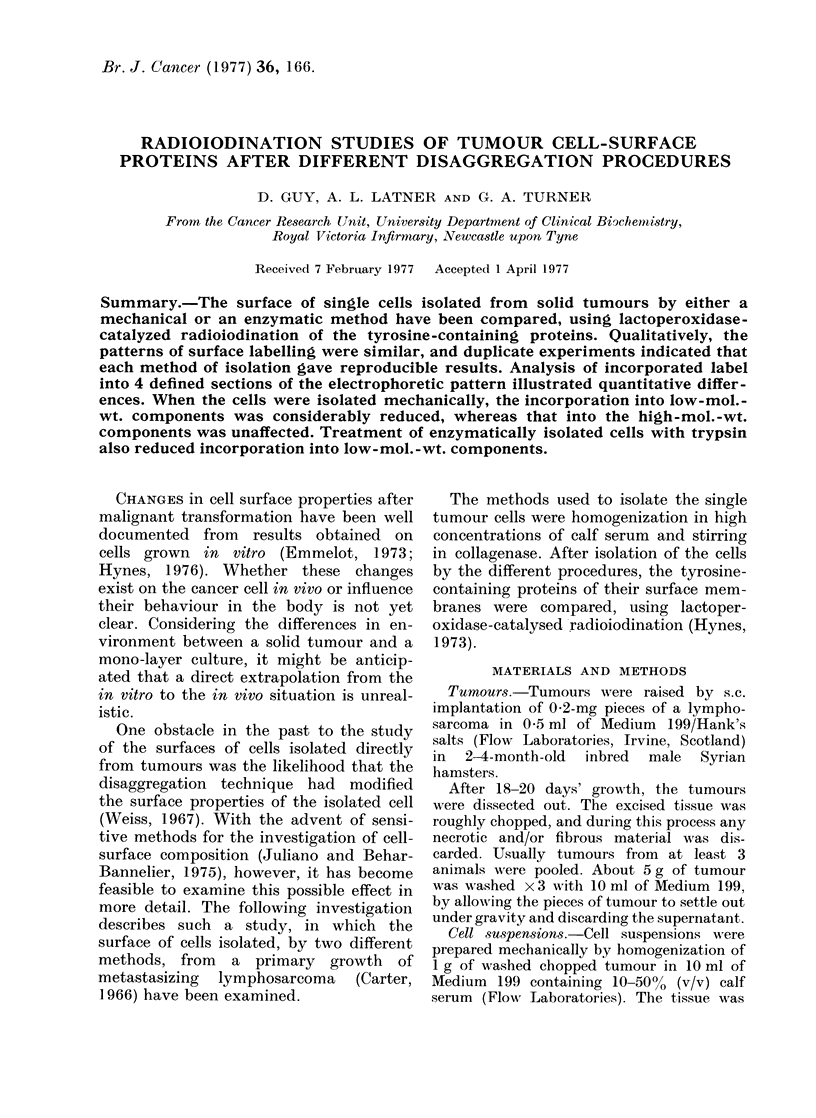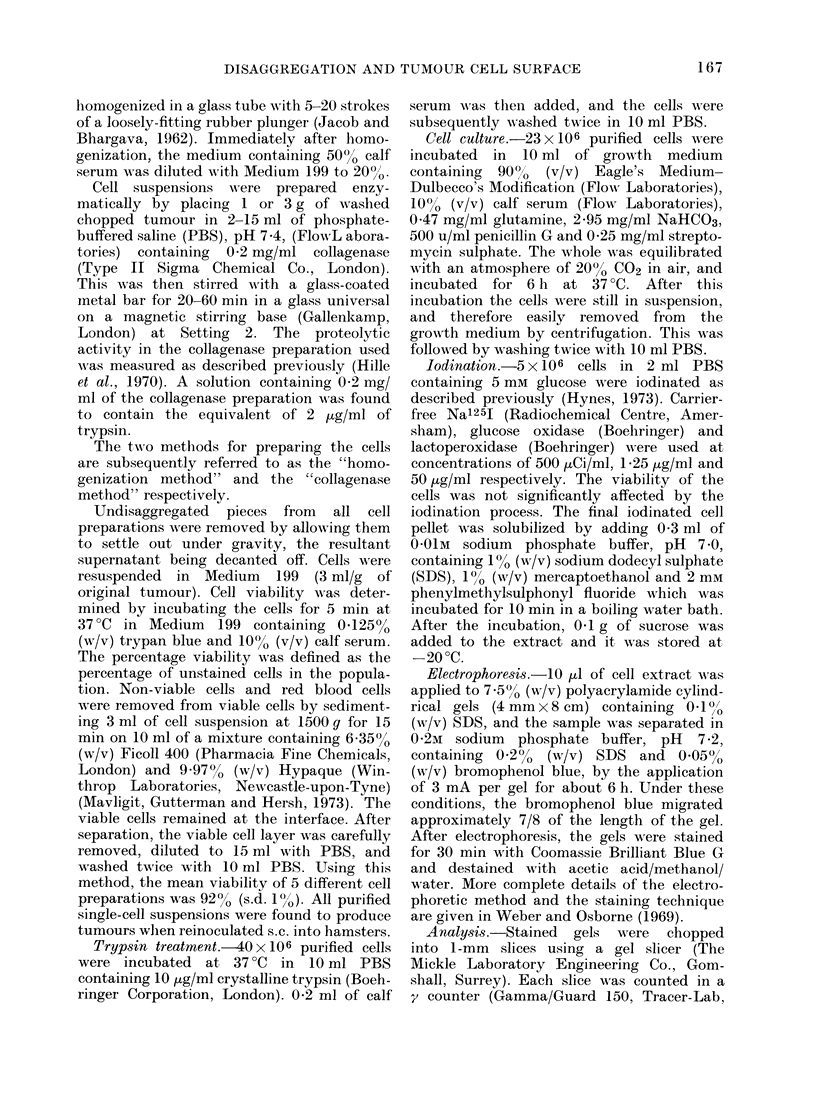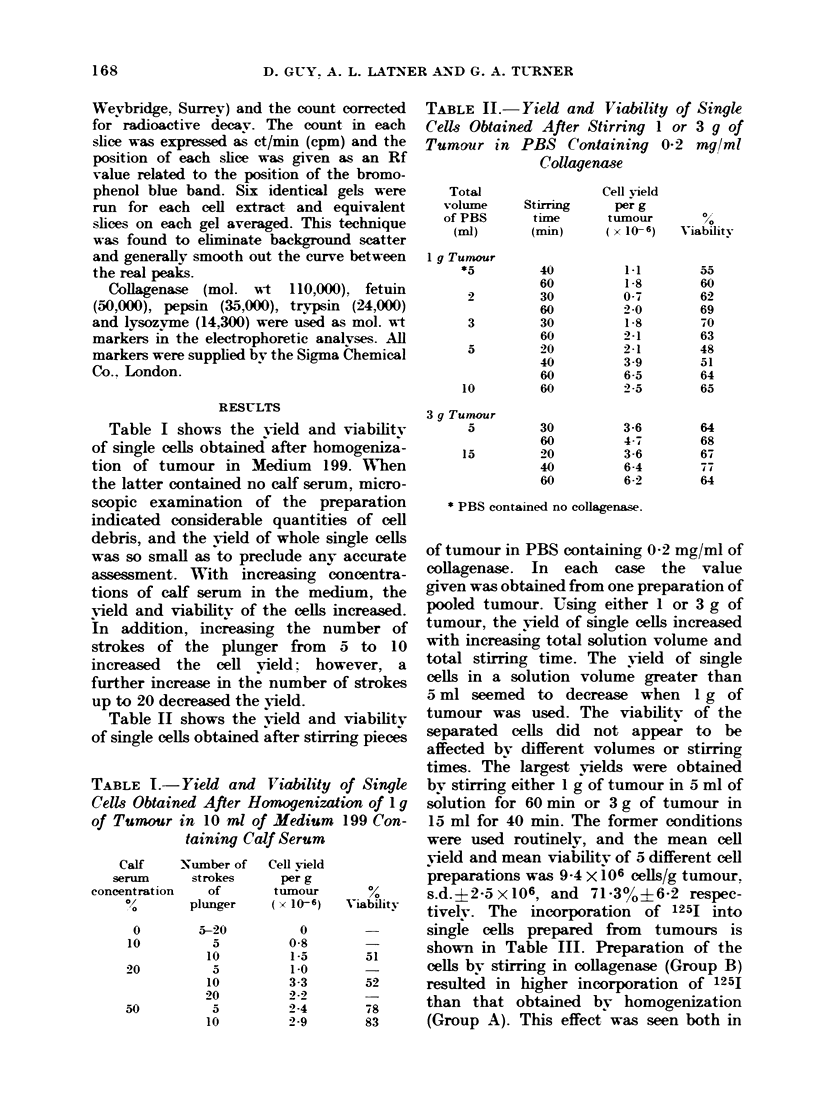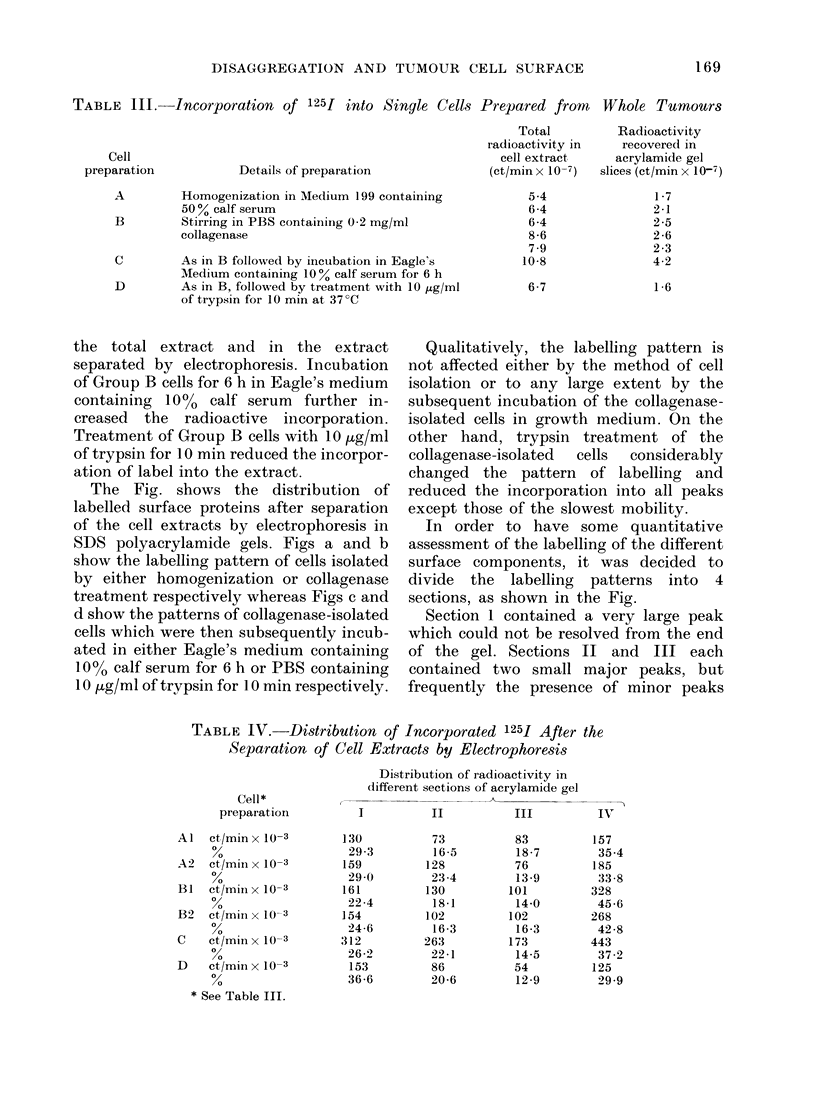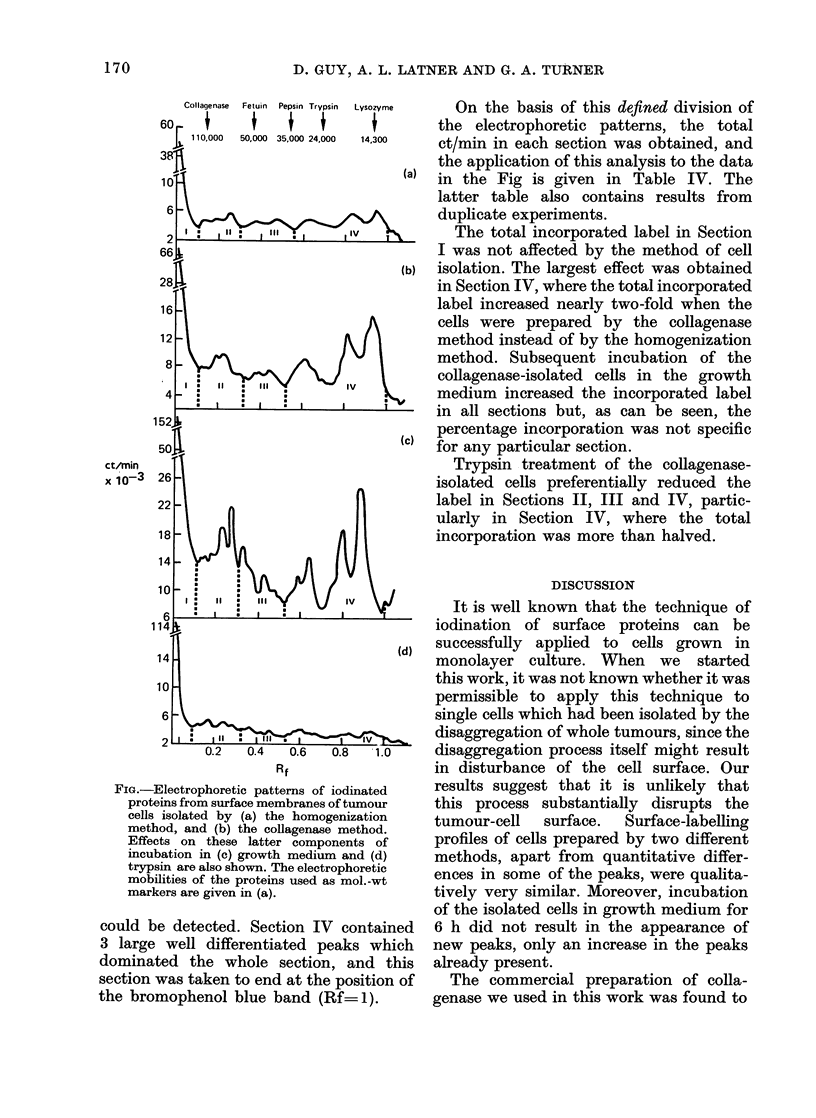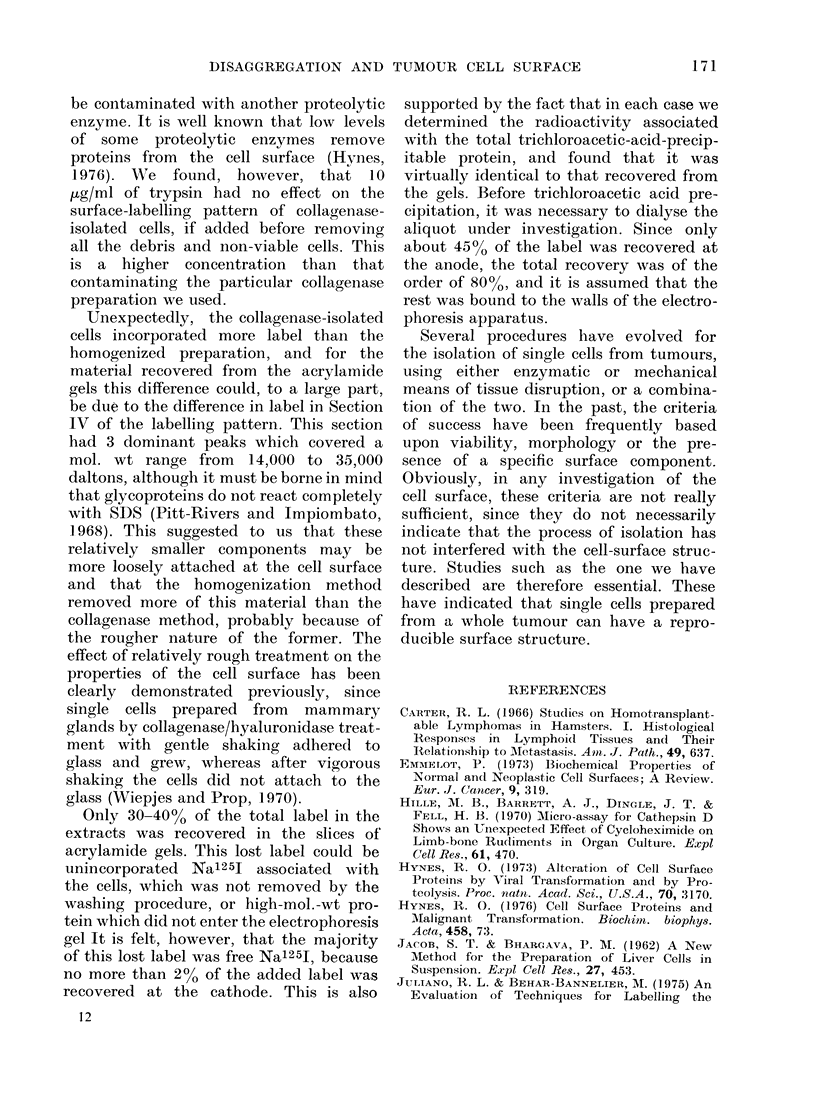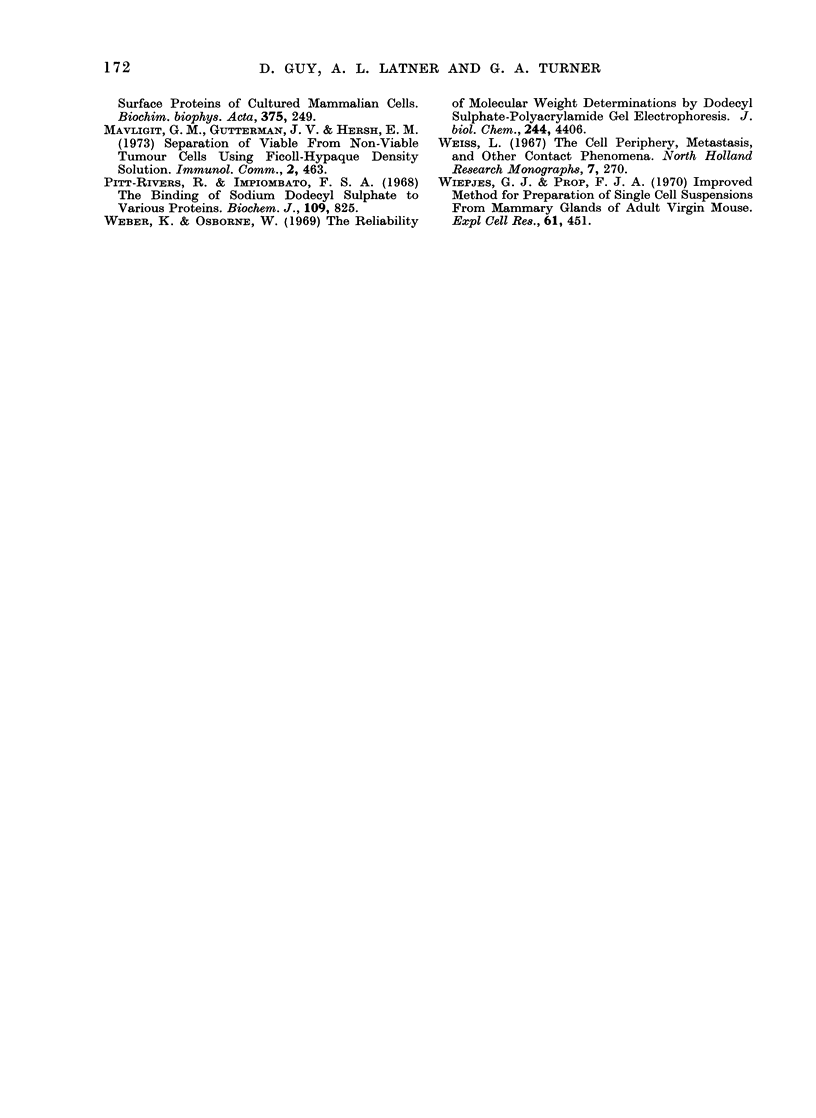# Radioiodination studies of tumour cell-surface proteins after different disaggregation procedures.

**DOI:** 10.1038/bjc.1977.173

**Published:** 1977-08

**Authors:** D. Guy, A. L. Latner, G. A. Turner

## Abstract

The surface of single cells isolated from solid tumours by either a mechanical or an enzymatic method have been compared, using lactoperoxidase-catalyzed radioiodination of the tyrosine-containing proteins. Qualitatively, the patterns of surface labelling were similar, and duplicate experiments indicated that each method of isolation gave reproducible results. Analysis of incorporated label into 4 defined sections of the electrophoretic pattern illustrated quantitative differences. When the cells were isolated mechanically, the incorporation into low-mol.- wt. components was considerably reduced, whereas that into the high-mol.-wt. components was unaffected. Treatment of enzymatically isolated cells with trypsin also reduced incorporation into low-mol.-wt components.


Br. J. Cancer (1977) 36, 166.

### RADIOIODINATION STUDIES OF TUMOUR CELL-SURFACE PROTEINS AFTER DIFFERENT DISAGGREGATION PROCEDURES

D. GUY, A. L. LATNER AND G. A. TURNER

*From the Cancer Research Unit, University Department of Clinical Biochemistry, Royal Victoria Infirmary, Newcastle upon Tyne*

Received 7 February 1977 Accepted 1 April 1977

**Summary.—The surface of single cells isolated from solid tumours by either a mechanical or an enzymatic method have been compared, using lactoperoxidase-catalyzed radioiodination of the tyrosine-containing proteins. Qualitatively, the patterns of surface labelling were similar, and duplicate experiments indicated that each method of isolation gave reproducible results. Analysis of incorporated label into 4 defined sections of the electrophoretic pattern illustrated quantitative differences. When the cells were isolated mechanically, the incorporation into low-mol.-wt. components was considerably reduced, whereas that into the high-mol.-wt. components was unaffected. Treatment of enzymatically isolated cells with trypsin also reduced incorporation into low-mol.-wt. components.**

CHANGES in cell surface properties after malignant transformation have been well documented from results obtained on cells grown *in vitro* (Emmelot, 1973; Hynes, 1976). Whether these changes exist on the cancer cell *in vivo* or influence their behaviour in the body is not yet clear. Considering the differences in environment between a solid tumour and a mono-layer culture, it might be anticipated that a direct extrapolation from the *in vitro* to the *in vivo* situation is unrealistic.

One obstacle in the past to the study of the surfaces of cells isolated directly from tumours was the likelihood that the disaggregation technique had modified the surface properties of the isolated cell (Weiss, 1967). With the advent of sensitive methods for the investigation of cell-surface composition (Juliano and Behar-Bannelier, 1975), however, it has become feasible to examine this possible effect in more detail. The following investigation describes such a study, in which the surface of cells isolated, by two different methods, from a primary growth of metastasizing lymphosarcoma (Carter, 1966) have been examined.

The methods used to isolate the single tumour cells were homogenization in high concentrations of calf serum and stirring in collagenase. After isolation of the cells by the different procedures, the tyrosine-containing proteins of their surface membranes were compared, using lactoperoxidase-catalysed radioiodination (Hynes, 1973).

### MATERIALS AND METHODS

*Tumours.*—Tumours were raised by s.c. implantation of 0·2-mg pieces of a lymphosarcoma in 0·5 ml of Medium 199/Hank's salts (Flow Laboratories, Irvine, Scotland) in 2–4-month-old inbred male Syrian hamsters.

After 18–20 days' growth, the tumours were dissected out. The excised tissue was roughly chopped, and during this process any necrotic and/or fibrous material was discarded. Usually tumours from at least 3 animals were pooled. About 5 g of tumour was washed ×3 with 10 ml of Medium 199, by allowing the pieces of tumour to settle out under gravity and discarding the supernatant.

*Cell suspensions.*—Cell suspensions were prepared mechanically by homogenization of 1 g of washed chopped tumour in 10 ml of Medium 199 containing 10–50% (v/v) calf serum (Flow Laboratories). The tissue was homogenized in a glass tube with 5–20 strokes of a loosely-fitting rubber plunger (Jacob and Bhargava, 1962). Immediately after homogenization, the medium containing 50% calf serum was diluted with Medium 199 to 20%.

Cell suspensions were prepared enzymatically by placing 1 or 3 g of washed chopped tumour in 2–15 ml of phosphate-buffered saline (PBS), pH 7·4, (FlowL aboratories) containing 0·2 mg/ml collagenase (Type II Sigma Chemical Co., London). This was then stirred with a glass-coated metal bar for 20–60 min in a glass universal on a magnetic stirring base (Gallenkamp, London) at Setting 2. The proteolytic activity in the collagenase preparation used was measured as described previously (Hille *et al.*, 1970). A solution containing 0·2 mg/ml of the collagenase preparation was found to contain the equivalent of 2 μg/ml of trypsin.

The two methods for preparing the cells are subsequently referred to as the "homogenization method" and the "collagenase method" respectively.

Undisaggregated pieces from all cell preparations were removed by allowing them to settle out under gravity, the resultant supernatant being decanted off. Cells were resuspended in Medium 199 (3 ml/g of original tumour). Cell viability was determined by incubating the cells for 5 min at 37 °C in Medium 199 containing 0·125% (w/v) trypan blue and 10% (v/v) calf serum. The percentage viability was defined as the percentage of unstained cells in the population. Non-viable cells and red blood cells were removed from viable cells by sedimenting 3 ml of cell suspension at 1500 *g* for 15 min on 10 ml of a mixture containing 6·35% (w/v) Ficoll 400 (Pharmacia Fine Chemicals, London) and 9·97% (w/v) Hypaque (Winthrop Laboratories, Newcastle-upon-Tyne) (Mavligit, Gutterman and Hersh, 1973). The viable cells remained at the interface. After separation, the viable cell layer was carefully removed, diluted to 15 ml with PBS, and washed twice with 10 ml PBS. Using this method, the mean viability of 5 different cell preparations was 92% (s.d. 1%). All purified single-cell suspensions were found to produce tumours when reinoculated s.c. into hamsters.

*Trypsin treatment.*—$40 \times 10^6$ purified cells were incubated at 37 °C in 10 ml PBS containing 10 μg/ml crystalline trypsin (Boehringer Corporation, London). 0·2 ml of calf serum was then added, and the cells were subsequently washed twice in 10 ml PBS.

*Cell culture.*—$23 \times 10^6$ purified cells were incubated in 10 ml of growth medium containing 90% (v/v) Eagle's Medium–Dulbecco's Modification (Flow Laboratories), 10% (v/v) calf serum (Flow Laboratories), 0·47 mg/ml glutamine, 2·95 mg/ml $NaHCO_3$, 500 u/ml penicillin G and 0·25 mg/ml streptomycin sulphate. The whole was equilibrated with an atmosphere of 20% $CO_2$ in air, and incubated for 6 h at 37 °C. After this incubation the cells were still in suspension, and therefore easily removed from the growth medium by centrifugation. This was followed by washing twice with 10 ml PBS.

*Iodination.*—$5 \times 10^6$ cells in 2 ml PBS containing 5 mM glucose were iodinated as described previously (Hynes, 1973). Carrier-free $Na^{125}I$ (Radiochemical Centre, Amersham), glucose oxidase (Boehringer) and lactoperoxidase (Boehringer) were used at concentrations of 500 μCi/ml, 1·25 μg/ml and 50 μg/ml respectively. The viability of the cells was not significantly affected by the iodination process. The final iodinated cell pellet was solubilized by adding 0·3 ml of 0·01M sodium phosphate buffer, pH 7·0, containing 1% (w/v) sodium dodecyl sulphate (SDS), 1% (w/v) mercaptoethanol and 2 mM phenylmethylsulphonyl fluoride which was incubated for 10 min in a boiling water bath. After the incubation, 0·1 g of sucrose was added to the extract and it was stored at −20 °C.

*Electrophoresis.*—10 μl of cell extract was applied to 7·5% (w/v) polyacrylamide cylindrical gels (4 mm × 8 cm) containing 0·1% (w/v) SDS, and the sample was separated in 0·2M sodium phosphate buffer, pH 7·2, containing 0·2% (w/v) SDS and 0·05% (w/v) bromophenol blue, by the application of 3 mA per gel for about 6 h. Under these conditions, the bromophenol blue migrated approximately 7/8 of the length of the gel. After electrophoresis, the gels were stained for 30 min with Coomassie Brilliant Blue G and destained with acetic acid/methanol/water. More complete details of the electrophoretic method and the staining technique are given in Weber and Osborne (1969).

*Analysis.*—Stained gels were chopped into 1-mm slices using a gel slicer (The Mickle Laboratory Engineering Co., Gomshall, Surrey). Each slice was counted in a γ counter (Gamma/Guard 150, Tracer-Lab,

Weybridge, Surrey) and the count corrected for radioactive decay. The count in each slice was expressed as ct/min (cpm) and the position of each slice was given as an Rf value related to the position of the bromophenol blue band. Six identical gels were run for each cell extract and equivalent slices on each gel averaged. This technique was found to eliminate background scatter and generally smooth out the curve between the real peaks.

Collagenase (mol. wt 110,000), fetuin (50,000), pepsin (35,000), trypsin (24,000) and lysozyme (14,300) were used as mol. wt markers in the electrophoretic analyses. All markers were supplied by the Sigma Chemical Co., London.

### RESULTS

Table I shows the yield and viability of single cells obtained after homogenization of tumour in Medium 199. When the latter contained no calf serum, microscopic examination of the preparation indicated considerable quantities of cell debris, and the yield of whole single cells was so small as to preclude any accurate assessment. With increasing concentrations of calf serum in the medium, the yield and viability of the cells increased. In addition, increasing the number of strokes of the plunger from 5 to 10 increased the cell yield; however, a further increase in the number of strokes up to 20 decreased the yield.

Table II shows the yield and viability of single cells obtained after stirring pieces of tumour in PBS containing 0·2 mg/ml of collagenase. In each case the value given was obtained from one preparation of pooled tumour. Using either 1 or 3 g of tumour, the yield of single cells increased with increasing total solution volume and total stirring time. The yield of single cells in a solution volume greater than 5 ml seemed to decrease when 1 g of tumour was used. The viability of the separated cells did not appear to be affected by different volumes or stirring times. The largest yields were obtained by stirring either 1 g of tumour in 5 ml of solution for 60 min or 3 g of tumour in 15 ml for 40 min. The former conditions were used routinely, and the mean cell yield and mean viability of 5 different cell preparations was $9 \cdot 4 \times 10^6$ cells/g tumour, s.d. $\pm 2 \cdot 5 \times 10^6$, and $71 \cdot 3\% \pm 6 \cdot 2$ respectively. The incorporation of $^{125}I$ into single cells prepared from tumours is shown in Table III. Preparation of the cells by stirring in collagenase (Group B) resulted in higher incorporation of $^{125}I$ than that obtained by homogenization (Group A). This effect was seen both in

TABLE I.—*Yield and Viability of Single Cells Obtained After Homogenization of* 1 g *of Tumour in* 10 *ml of Medium* 199 *Containing Calf Serum*

| Calf serum concentration % | Number of strokes of plunger | Cell yield per g tumour ($\times 10^{-6}$) | % Viability |
|---|---|---|---|
| 0 | 5–20 | 0 | — |
| 10 | 5 | 0·8 | — |
| | 10 | 1·5 | 51 |
| 20 | 5 | 1·0 | — |
| | 10 | 3·3 | 52 |
| | 20 | 2·2 | — |
| 50 | 5 | 2·4 | 78 |
| | 10 | 2·9 | 83 |

TABLE II.—*Yield and Viability of Single Cells Obtained After Stirring* 1 *or* 3 *g of Tumour in PBS Containing* 0·2 *mg/ml Collagenase*

| Total volume of PBS (ml) | Stirring time (min) | Cell yield per g tumour ($\times 10^{-6}$) | % Viability |
|---|---|---|---|
| 1 *g Tumour* | | | |
| *5 | 40 | 1·1 | 55 |
| | 60 | 1·8 | 60 |
| 2 | 30 | 0·7 | 62 |
| | 60 | 2·0 | 69 |
| 3 | 30 | 1·8 | 70 |
| | 60 | 2·1 | 63 |
| 5 | 20 | 2·1 | 48 |
| | 40 | 3·9 | 51 |
| | 60 | 6·5 | 64 |
| 10 | 60 | 2·5 | 65 |
| 3 *g Tumour* | | | |
| 5 | 30 | 3·6 | 64 |
| | 60 | 4·7 | 68 |
| 15 | 20 | 3·6 | 67 |
| | 40 | 6·4 | 77 |
| | 60 | 6·2 | 64 |

* PBS contained no collagenase.

TABLE III.—*Incorporation of $^{125}I$ into Single Cells Prepared from Whole Tumours*

| Cell preparation | Details of preparation | Total radioactivity in cell extract (ct/min × $10^{-7}$) | Radioactivity recovered in acrylamide gel slices (ct/min × $10^{-7}$) |
|---|---|---|---|
| A | Homogenization in Medium 199 containing | 5·4 | 1·7 |
| | 50% calf serum | 6·4 | 2·1 |
| B | Stirring in PBS containing 0·2 mg/ml | 6·4 | 2·5 |
| | collagenase | 8·6 | 2·6 |
| | | 7·9 | 2·3 |
| C | As in B followed by incubation in Eagle's Medium containing 10% calf serum for 6 h | 10·8 | 4·2 |
| D | As in B, followed by treatment with 10 μg/ml of trypsin for 10 min at 37 °C | 6·7 | 1·6 |

the total extract and in the extract separated by electrophoresis. Incubation of Group B cells for 6 h in Eagle's medium containing 10% calf serum further increased the radioactive incorporation. Treatment of Group B cells with 10 μg/ml of trypsin for 10 min reduced the incorporation of label into the extract.

The Fig. shows the distribution of labelled surface proteins after separation of the cell extracts by electrophoresis in SDS polyacrylamide gels. Figs a and b show the labelling pattern of cells isolated by either homogenization or collagenase treatment respectively whereas Figs c and d show the patterns of collagenase-isolated cells which were then subsequently incubated in either Eagle's medium containing 10% calf serum for 6 h or PBS containing 10 μg/ml of trypsin for 10 min respectively.

Qualitatively, the labelling pattern is not affected either by the method of cell isolation or to any large extent by the subsequent incubation of the collagenase-isolated cells in growth medium. On the other hand, trypsin treatment of the collagenase-isolated cells considerably changed the pattern of labelling and reduced the incorporation into all peaks except those of the slowest mobility.

In order to have some quantitative assessment of the labelling of the different surface components, it was decided to divide the labelling patterns into 4 sections, as shown in the Fig.

Section 1 contained a very large peak which could not be resolved from the end of the gel. Sections II and III each contained two small major peaks, but frequently the presence of minor peaks

TABLE IV.—*Distribution of Incorporated $^{125}I$ After the Separation of Cell Extracts by Electrophoresis*

| Cell* preparation | | Distribution of radioactivity in different sections of acrylamide gel | | | |
|---|---|---|---|---|---|
| | | I | II | III | IV |
| A1 | ct/min × $10^{-3}$ | 130 | 73 | 83 | 157 |
| | % | 29·3 | 16·5 | 18·7 | 35·4 |
| A2 | ct/min × $10^{-3}$ | 159 | 128 | 76 | 185 |
| | % | 29·0 | 23·4 | 13·9 | 33·8 |
| B1 | ct/min × $10^{-3}$ | 161 | 130 | 101 | 328 |
| | % | 22·4 | 18·1 | 14·0 | 45·6 |
| B2 | ct/min × $10^{-3}$ | 154 | 102 | 102 | 268 |
| | % | 24·6 | 16·3 | 16·3 | 42·8 |
| C | ct/min × $10^{-3}$ | 312 | 263 | 173 | 443 |
| | % | 26·2 | 22·1 | 14·5 | 37·2 |
| D | ct/min × $10^{-3}$ | 153 | 86 | 54 | 125 |
| | % | 36·6 | 20·6 | 12·9 | 29·9 |

* See Table III.

FIG.—Electrophoretic patterns of iodinated proteins from surface membranes of tumour cells isolated by (a) the homogenization method, and (b) the collagenase method. Effects on these latter components of incubation in (c) growth medium and (d) trypsin are also shown. The electrophoretic mobilities of the proteins used as mol.-wt markers are given in (a).

could be detected. Section IV contained 3 large well differentiated peaks which dominated the whole section, and this section was taken to end at the position of the bromophenol blue band (Rf=1).

On the basis of this *defined* division of the electrophoretic patterns, the total ct/min in each section was obtained, and the application of this analysis to the data in the Fig is given in Table IV. The latter table also contains results from duplicate experiments.

The total incorporated label in Section I was not affected by the method of cell isolation. The largest effect was obtained in Section IV, where the total incorporated label increased nearly two-fold when the cells were prepared by the collagenase method instead of by the homogenization method. Subsequent incubation of the collagenase-isolated cells in the growth medium increased the incorporated label in all sections but, as can be seen, the percentage incorporation was not specific for any particular section.

Trypsin treatment of the collagenase-isolated cells preferentially reduced the label in Sections II, III and IV, particularly in Section IV, where the total incorporation was more than halved.

### DISCUSSION

It is well known that the technique of iodination of surface proteins can be successfully applied to cells grown in monolayer culture. When we started this work, it was not known whether it was permissible to apply this technique to single cells which had been isolated by the disaggregation of whole tumours, since the disaggregation process itself might result in disturbance of the cell surface. Our results suggest that it is unlikely that this process substantially disrupts the tumour-cell surface. Surface-labelling profiles of cells prepared by two different methods, apart from quantitative differences in some of the peaks, were qualitatively very similar. Moreover, incubation of the isolated cells in growth medium for 6 h did not result in the appearance of new peaks, only an increase in the peaks already present.

The commercial preparation of collagenase we used in this work was found to be contaminated with another proteolytic enzyme. It is well known that low levels of some proteolytic enzymes remove proteins from the cell surface (Hynes, 1976). We found, however, that 10 μg/ml of trypsin had no effect on the surface-labelling pattern of collagenase-isolated cells, if added before removing all the debris and non-viable cells. This is a higher concentration than that contaminating the particular collagenase preparation we used.

Unexpectedly, the collagenase-isolated cells incorporated more label than the homogenized preparation, and for the material recovered from the acrylamide gels this difference could, to a large part, be due to the difference in label in Section IV of the labelling pattern. This section had 3 dominant peaks which covered a mol. wt range from 14,000 to 35,000 daltons, although it must be borne in mind that glycoproteins do not react completely with SDS (Pitt-Rivers and Impiombato, 1968). This suggested to us that these relatively smaller components may be more loosely attached at the cell surface and that the homogenization method removed more of this material than the collagenase method, probably because of the rougher nature of the former. The effect of relatively rough treatment on the properties of the cell surface has been clearly demonstrated previously, since single cells prepared from mammary glands by collagenase/hyaluronidase treatment with gentle shaking adhered to glass and grew, whereas after vigorous shaking the cells did not attach to the glass (Wiepjes and Prop, 1970).

Only 30–40% of the total label in the extracts was recovered in the slices of acrylamide gels. This lost label could be unincorporated $Na^{125}I$ associated with the cells, which was not removed by the washing procedure, or high-mol.-wt protein which did not enter the electrophoresis gel It is felt, however, that the majority of this lost label was free $Na^{125}I$, because no more than 2% of the added label was recovered at the cathode. This is also supported by the fact that in each case we determined the radioactivity associated with the total trichloroacetic-acid-precipitable protein, and found that it was virtually identical to that recovered from the gels. Before trichloroacetic acid precipitation, it was necessary to dialyse the aliquot under investigation. Since only about 45% of the label was recovered at the anode, the total recovery was of the order of 80%, and it is assumed that the rest was bound to the walls of the electrophoresis apparatus.

Several procedures have evolved for the isolation of single cells from tumours, using either enzymatic or mechanical means of tissue disruption, or a combination of the two. In the past, the criteria of success have been frequently based upon viability, morphology or the presence of a specific surface component. Obviously, in any investigation of the cell surface, these criteria are not really sufficient, since they do not necessarily indicate that the process of isolation has not interfered with the cell-surface structure. Studies such as the one we have described are therefore essential. These have indicated that single cells prepared from a whole tumour can have a reproducible surface structure.

### REFERENCES

Carter, R. L. (1966) Studies on Homotransplantable Lymphomas in Hamsters. I. Histological Responses in Lymphoid Tissues and Their Relationship to Metastasis. *Am. J. Path.*, **49**, 637.

Emmelot, P. (1973) Biochemical Properties of Normal and Neoplastic Cell Surfaces; A Review. *Eur. J. Cancer*, **9**, 319.

Hille, M. B., Barrett, A. J., Dingle, J. T. & Fell, H. B. (1970) Micro-assay for Cathepsin D Shows an Unexpected Effect of Cycloheximide on Limb-bone Rudiments in Organ Culture. *Expl Cell Res.*, **61**, 470.

Hynes, R. O. (1973) Alteration of Cell Surface Proteins by Viral Transformation and by Proteolysis. *Proc. natn. Acad. Sci., U.S.A.*, **70**, 3170.

Hynes, R. O. (1976) Cell Surface Proteins and Malignant Transformation. *Biochim. biophys. Acta*, **458**, 73.

Jacob, S. T. & Bhargava, P. M. (1962) A New Method for the Preparation of Liver Cells in Suspension. *Expl Cell Res.*, **27**, 453.

Juliano, R. L. & Behar-Bannelier, M. (1975) An Evaluation of Techniques for Labelling the

Surface Proteins of Cultured Mammalian Cells. *Biochim. biophys. Acta*, **375**, 249.
MAVLIGIT, G. M., GUTTERMAN, J. V. & HERSH, E. M. (1973) Separation of Viable From Non-Viable Tumour Cells Using Ficoll-Hypaque Density Solution. *Immunol. Comm.*, **2**, 463.
PITT-RIVERS, R. & IMPIOMBATO, F. S. A. (1968) The Binding of Sodium Dodecyl Sulphate to Various Proteins. *Biochem. J.*, **109**, 825.
WEBER, K. & OSBORNE, W. (1969) The Reliability of Molecular Weight Determinations by Dodecyl Sulphate-Polyacrylamide Gel Electrophoresis. *J. biol. Chem.*, **244**, 4406.
WEISS, L. (1967) The Cell Periphery, Metastasis, and Other Contact Phenomena. *North Holland Research Monographs*, **7**, 270.
WIEPJES, G. J. & PROP, F. J. A. (1970) Improved Method for Preparation of Single Cell Suspensions From Mammary Glands of Adult Virgin Mouse. *Expl Cell Res.*, **61**, 451.